# DTI and VBM reveal white matter changes without associated gray matter changes in patients with idiopathic restless legs syndrome

**DOI:** 10.1002/brb3.327

**Published:** 2015-07-30

**Authors:** Marcus Belke, Johannes T Heverhagen, Boris Keil, Felix Rosenow, Wolfgang H Oertel, Karin Stiasny-Kolster, Susanne Knake, Katja Menzler

**Affiliations:** 1 Department of Neurology, Philipps-University MarburgBaldingerstrasse, Marburg, 35043, Germany; 2 Department of Diagnostic Radiology, Philipps-University MarburgBaldingerstrasse, Marburg, 35043, Germany; 3 Epilepsy Center Franfurt Rhein-Main, Department of Neurology, Johann Wolfgang Goethe UniversityFrankfurt am Main, Germany; 4 Somnomar, Institute for Medical Research and Sleep Medicine MarburgMarburger Strasse 9a, Marburg, 35043, Germany

**Keywords:** Diffusion tensor imaging, gray matter changes, restless legs syndrome, voxel-based morphometry, white matter changes

## Abstract

**Background and Purpose:**

We evaluated cerebral white and gray matter changes in patients with iRLS in order to shed light on the pathophysiology of this disease.

**Methods:**

Twelve patients with iRLS were compared to 12 age- and sex-matched controls using whole-head diffusion tensor imaging (DTI) and voxel-based morphometry (VBM) techniques. Evaluation of the DTI scans included the voxelwise analysis of the fractional anisotropy (FA), radial diffusivity (RD), and axial diffusivity (AD).

**Results:**

Diffusion tensor imaging revealed areas of altered FA in subcortical white matter bilaterally, mainly in temporal regions as well as in the right internal capsule, the pons, and the right cerebellum. These changes overlapped with changes in RD. Voxel-based morphometry did not reveal any gray matter alterations.

**Conclusions:**

We showed altered diffusion properties in several white matter regions in patients with iRLS. White matter changes could mainly be attributed to changes in RD, a parameter thought to reflect altered myelination. Areas with altered white matter microstructure included areas in the internal capsule which include the corticospinal tract to the lower limbs, thereby supporting studies that suggest changes in sensorimotor pathways associated with RLS.

## Background and Purpose

Idiopathic restless legs syndrome (iRLS) is a common neurological disorder characterized by an urge to move the legs, which is usually accompanied or caused by an unpleasant sensation in the legs which increases in intensity at rest and in the evening or night and is relieved by movement (Allen et al. 2003).

Proposed underlying pathophysiological mechanisms include a primary subcortical involvement of the thalamus, cerebellum, brainstem, and spinal cord (Bucher et al. 1997; Etgen et al. 2005; Unrath and Kassubek 2006; Paulus et al. 2007; Margariti et al. 2012), involvement of the dopaminergic (Staedt et al. 1993, 1995a; Turjanski et al. 1999; Ruottinen et al. 2000; Michaud et al. 2002; Cervenka et al. 2006; Margariti et al. 2012) and opioidergic system (von Spiczak et al. 2005), the sensorimotor network (Unrath et al. 2007, 2008; Margariti et al. 2012), and changes in brain iron concentration (Allen et al. 2001; Astrakas et al. 2008).

Several neuroimaging techniques including voxel-based morphometry (VBM, Etgen et al. 2005; Unrath et al. 2007; Connor et al. 2010; Hornyak et al. 2007), diffusion tensor imaging (DTI, Unrath et al. 2008), functional magnetic resonance imaging (fMRI, Bucher et al. 1997; Margariti et al. 2012; Astrakas et al. 2008; Spiegelhalder et al. 2008), positron emission tomography (PET, Turjanski et al. 1999; Ruottinen et al. 2000; Cervenka et al. 2006; von Spiczak et al. 2005), and single photon emission computed tomography (SPECT, Michaud et al. 2002; Staedt et al. 1993, 1995a) support these pathophysiological considerations by showing changes in the thalamus (Bucher et al. 1997; Etgen et al. 2005; Astrakas et al. 2008; Unrath et al. 2008; Margariti et al. 2012), cerebellum (Bucher et al. 1997; Margariti et al. 2012), brainstem (Bucher et al. 1997), basal ganglia or dopaminergic system (Staedt et al. 1993, 1995a; Turjanski et al. 1999; Ruottinen et al. 2000; Michaud et al. 2002; Cervenka et al. 2006; Astrakas et al. 2008), premotor, primary or higher order motor or somatosensory brain regions (Unrath et al. 2007, 2008; Margariti et al. 2012), and the limbic system (Astrakas et al. 2008; Margariti et al. 2012). However, the results remain conflicting and numerous other studies show no brain changes in patients with idiopathic RLS using diverse neuroimaging techniques (Trenkwalder et al. 1999; Eisensehr et al. 2001; Tribl et al. 2004; Celle et al. 2010; Comley et al. 2012; Rizzo et al. 2012) or histological evaluation (Earley et al. 2009).

Diffusion tensor imaging is an MRI technique that enables noninvasive, in vivo visualization of white matter microstructural or functional changes. Voxel-based morphometry offers the opportunity to investigate subtle changes in gray matter volume. Both techniques have been applied successfully to several neurodegenerative disorders, providing insight in the underlying pathophysiology of these conditions (Kaufmann et al. 2002; Brenneis et al. 2005; Salat et al. 2008; Knake et al. 2010a; Unger et al. 2010; Menzler et al. 2011, 2012).

The aim of this study was to investigate the changes of white matter diffusion parameters and gray matter volume in the brains of patients with iRLS using whole-head DTI and VBM combined with a hypothesis-free analysis approach in order to gain information on the pathophysiology of iRLS.

## Patients and Methods

### Patients

Twelve patients fulfilling the International Restless Legs Syndrome Study Group (IRLSSG) diagnostic criteria for iRLS (Allen et al. 2003) and 12 age- and sex-matched healthy control subjects were included in the study. All patients were recruited in our outpatient clinic, where secondary forms of RLS were excluded. The iRLS group consisted of nine women and three men with a mean age of 58.5 (±8.35) years and a mean disease duration of 12.1 (±8.74) years. Four of the 12 patients had a positive family history for RLS. Five patients were treated with L-dopa and three with pramipexole at the time of image acquisition. One patient had discontinued lisuride in 2007 and the remaining three patients were drug naïve. Clinical Global Impression ratings (CGI; 1 = normal to 6 = very severe) revealed that six patients suffered from moderate RLS (CGI = 4) and six patients from severe RLS (CGI = 5).

None of the patients showed evidence of additional neurological or psychiatric diseases on neurological examination. Iron, ferritin, transferrin saturation, and soluble transferrin receptor were within the normal range.

The control subjects (nine women, three men) had no history of RLS or other neurological or sleep disorders and showed no abnormalities in the neurological examination. Their mean age was 56.8 (±10.1) years.

The study was approved by the local IRB. All patients and healthy subjects gave written informed consent to participate in the study.

### MRI acquisition

The DTI scans were collected on a Siemens 1.5T Sonata MRI scanner (Siemens Medical Solutions, Erlangen, Germany) using a cp head array coil. A single shot echo planar sequence with a twice-refocused spin echo pulse, optimized to minimize eddy current-induced image distortions, was performed with the following parameters: TR/TE = 10600/104 ms, flip angle = 90°, *b* = 1000 s mm^2^, 128 × 128 mm FOV, voxel size 2 × 2 × 2.4 mm. Five T2 b0 images and 30 DWI b1000 images were collected during one scan. A 3D T1 magnetization prepared rapid gradient echo sequence (MPRAGE) was acquired during the same session for VBM (image parameters: TR/TE = 1480/3.04 ms, flip angle = 15°, 512 × 512 mm FOV, voxel size 0.4883 × 0.4883 × 1 mm). All images were investigated to be free of motion or ghosting, high frequency and/or wrap-around artifacts at the time of image acquisition.

### MRI analysis

For DTI and VBM calculations, we used programs published by FSL (Good et al. 2001; Smith et al. 2004; Andersson et al. 2007; Douaud et al. 2007).

### DTI

#### DTI preprocessing and analysis

Image preprocessing was performed as described previously (Menzler et al. 2011). Diffusion volumes were motion-corrected and averaged using FLIRT with mutual information cost function to register each direction to the minimally eddy current distorted T2-weighted b0 DTI volume that had no diffusion weighting. Eigenvalues (*λ*_1_, *λ*_2_, *λ*_3_) and eigenvectors of the diffusion tensor matrix for each voxel were computed from the DTI volumes for each subject on a voxel-by-voxel basis using conventional reconstruction methods. These tools are included in the FreeSurfer package (FreeSurfer version 4.2.0; http://surfer.nmr.mgh.harvard.edu/).

#### Fractional anisotropy and diffusivity map calculation

Brain tissue integrity was assessed using DTI measures of fractional anisotropy (FA), axial diffusivity (AD), and radial diffusivity (RD) as described previously (Menzler et al. 2011). The primary measure acquired from the DTI data was the fractional anisotropy (FA), a scalar metric unit describing the directionality of water diffusion. FA is dependent on the orientational coherence of the diffusion compartments within a voxel and reflects the degree of tissue organization or alignment (Pierpaoli and Basser 1996). The use of this parameter is described in a number of recent studies of tissue deterioration. FA was calculated using the standard formula defined previously (Basser 1997). To further characterize tissue organization, we additionally examined measures of axial diffusivity (AD, *λ*_1_) and radial diffusivity (RD, [*λ*_2_ + *λ*_3_]/2). Axial diffusivity measures the diffusivity along the primary diffusion direction and is assumed to contribute information regarding the integrity of axons (Glenn et al. 2003) or changes in extra-axonal/extracellular space (Beaulieu and Allen 1994). Radial diffusivity represents the diffusivities along directions that are orthogonal to the primary diffusion direction and is assumed to characterize changes associated with myelination or glial cell morphology (Song et al. 2002, 2003). T2 b0 images were obtained using the exact parameters as the diffusion-sensitive images except without any diffusion weighting. Those images were analyzed to determine whether changes other than those in tissue microstructure contributed to the observed effects, such as technical artifacts or individual large scale signal changes such as WM signal abnormalities (e.g. hyperintensities).

#### Nonlinear registration and tract-based spatial statistics (TBSS)

Voxelwise statistical analysis of the FA data was carried out using TBSS, which is part of the FSL data analysis suite. First, the brains were extracted from T2 b0 images using a brain extraction tool (BET). Those extracted brains were used to mask the brain on the FA images. The extracted brains were fed into the FSL TBSS processing stream (http://www.fmrib.ox.ac.uk/fsl/tbss/index.html). All subjects’ masked FA data were registered to the FMRIB 58 brain. First all brains underwent a linear coregistration to the standard space using the tool FLIRT, then all brains were coregistered using the nonlinear registration tool FNIRT, which uses a b-spline representation of the registration warp field. After the registration to the FMRIB 58 brain, all brains were transformed into the MNI 152 space. Next, a mean FA image was created and thinned to create a mean FA skeleton which represents the centers of all tracts the group has in common. Each subject’s aligned FA data were then projected onto this skeleton. Data along the skeleton were smoothed utilizing an anatomical constraint to limit the smoothing to neighbouring data within adjacent voxels along the skeleton. For smoothing the neighbouring voxels within a cube of 6 mm edge length were used to calculate the mean. The smoothing step was performed using matlab (Matlab 7.6.0.324 (R2008a), MathWorks, Aachen, Germany). All analyses were masked to only display regions with FA values of >0.2 as an additional procedure to avoid examination of regions that are likely comprised of multiple tissue types or fiber orientations. The exact transformations derived for the anisotropy maps were applied to the axial and radial diffusivity volumes for matched processing of all image volumes.

#### Group analysis

The resulting skeletonized images were fed into voxelwise cross-subject statistics. Cross-subject statistics were applied to analyze differences in FA, AD, and RD between patients with RLS and normal controls. For the group analysis, we used the tool mri_glmfit of the FreeSurfer package. The data were fit into a generalized linear model and an unpaired t test was performed. The resulting data were corrected for multiple comparisons by a permutation-based approach. Therefore, 12,000 simulations were performed under the null hypothesis. This approach was based on the AFNI null-z simulator (AlphaSim; http://afni.nimh.nih.gov/afni/doc/manual/AlphaSim). Last, the data were clustered. We chose a minimum value of *P* = 0.01 for the cluster calculation. To display the results, all figures were made with exactly the same parameters, showing clusters with a significance of *P* < 0.01. The significance was given as the negative decadic logarithm of the *P*-value (*P* = 10 − *x*). A blue – light blue color indicated clusters, where the measured FA was significantly decreased in the RLS group compared with controls, whereas the red to yellow color indicated a significant increase of the FA. For visualization purposes, clusters were dilated, using the mean dilation method of fslmath (a tool included in the FSL stream).

### VBM

Structural data were analyzed with the FSL-VBM processing stream (Smith et al. 2004; Douaud et al. 2007). This processing stream uses an optimized VBM protocol described previously (Good et al. 2001).

First, structural images were brain extracted using the FSL tool BET and gray matter-segmented before being registered to the MNI 152 standard space using FNIRT for nonlinear registration (Andersson et al. 2007).

The resulting images were averaged and flipped along the *x*-axis to create a left-right symmetric, study-specific gray matter template. Second, all native gray matter images were nonlinearly registered to this study-specific template and “modulated” to correct for local expansion (or contraction) due to the nonlinear component of the spatial transformation.

The modulated gray matter images were then smoothed with an isotropic Gaussian kernel with a sigma of 3 mm.

Finally, voxelwise GLM was applied using permutation-based nonparametric testing, correcting for multiple comparisons across space. Therefore, we used the fsl tool randomize and performed 500,000 simulations under the null hypothesis. The other parameters were chosen as described in the DTI section.

## Results

### DTI results

Diffusion tensor imaging revealed areas of altered FA in subcortical white matter bilaterally, mainly in temporal regions, as well as in the right internal capsule in areas containing the pyramidal tract to the lower limb, the pons, and right cerebellum (Table 2001, Fig.1). Further evaluation using radial and axial diffusivity showed changes in radial diffusivity in patients with RLS in small areas in the subcortical white matter, the pons and the cerebellum bilaterally (Table 2003, Fig.2). Small areas of altered axial diffusivity were located in the subcortical white matter, the left internal capsule, and the right cerebellum (Table 2003).

**Table 1:** Brain regions showing significant differences in FA values in patients with RLS as compared to healthy controls

Region	Size (mm^3^)	Maximum *P* (10^−x^)	Corrected Cluster wise *P*	*X*	*Y*	*Z*
Regions with significant lower FA values						
Temporal (R)	80	−2.38	<0.0001	32	85	106
Brainstem	14	−2.04	<0.0001	89	97	44
Olfactory	48	−2.03	<0.0001	95	143	54
Cerebellum	212	−2.84	<0.0001	54	54	33
Regions with significant higher FA values						
Temporal (R)	382	2.80	<0.0001	49	139	41
Temporal (L)	133	2.85	<0.0001	140	139	56
Lingula/occipital	95	3.14	<0.0001	68	65	71
Capsula int. (R)	89	2.24	<0.0001	66	115	84

FA, Fractional anisotropy; RLS, Restless legs syndrome.

The *P*-values given are clusterwise *P*-values. Several connected voxels with significant changes were automatically searched and merged into one cluster. The size of each cluster is given in mm^3^. *X*, *Y,* and *Z* coordinates are presented in MNI 152 space.

**Table 2:** Brain regions showing significant differences in RD and AD values in patients with RLS as compared to healthy controls

Region	Size (mm^3^)	Maximum *P* (10^−x^)	Corrected Cluster wise *P*	*X*	*Y*	*Z*
Regions with significant higher RD values						
Pons	54	2.46	<0.0001	94	102	41
Cerebellum left	60	2.66	<0.0001	100	48	32
Cerebellum right	155	2.86	<0.0001	59	49	34
Subcortical right	89	3.97	<0.0001	31	113	96
Subcortical right	38	1.97	<0.0001	32	86	106
Subcortical right	7	1.85	<0.0001	42	126	92
Subcortical left	14	1.99	<0.0001	102	98	128
Parietal right	67	2.59	<0.0001	63	77	77
Callosal body	15	2.07	<0.0001	70	79	90
Regions with significant higher AD values						
Crus cerebri right	37	−3.18	<0.0001	103	117	62
Pallidum right	16	−2.27	<0.0001	75	138	61
Cerebellum right	35	−2.35	<0.0001	44	72	37
Subcortical right	58	−2.09	<0.0001	133	92	78

AD, Axial diffusivity; RD, Radial diffusivity; RLS, Restless legs syndrome.

The *P*-values given are clusterwise *P*-values. Several connected voxels with significant changes were automatically searched and merged into one cluster. The size of each cluster is given in mm^3^. *X*, *Y,* and *Z* coordinates are presented in MNI 152 space.

**Figure 1:**
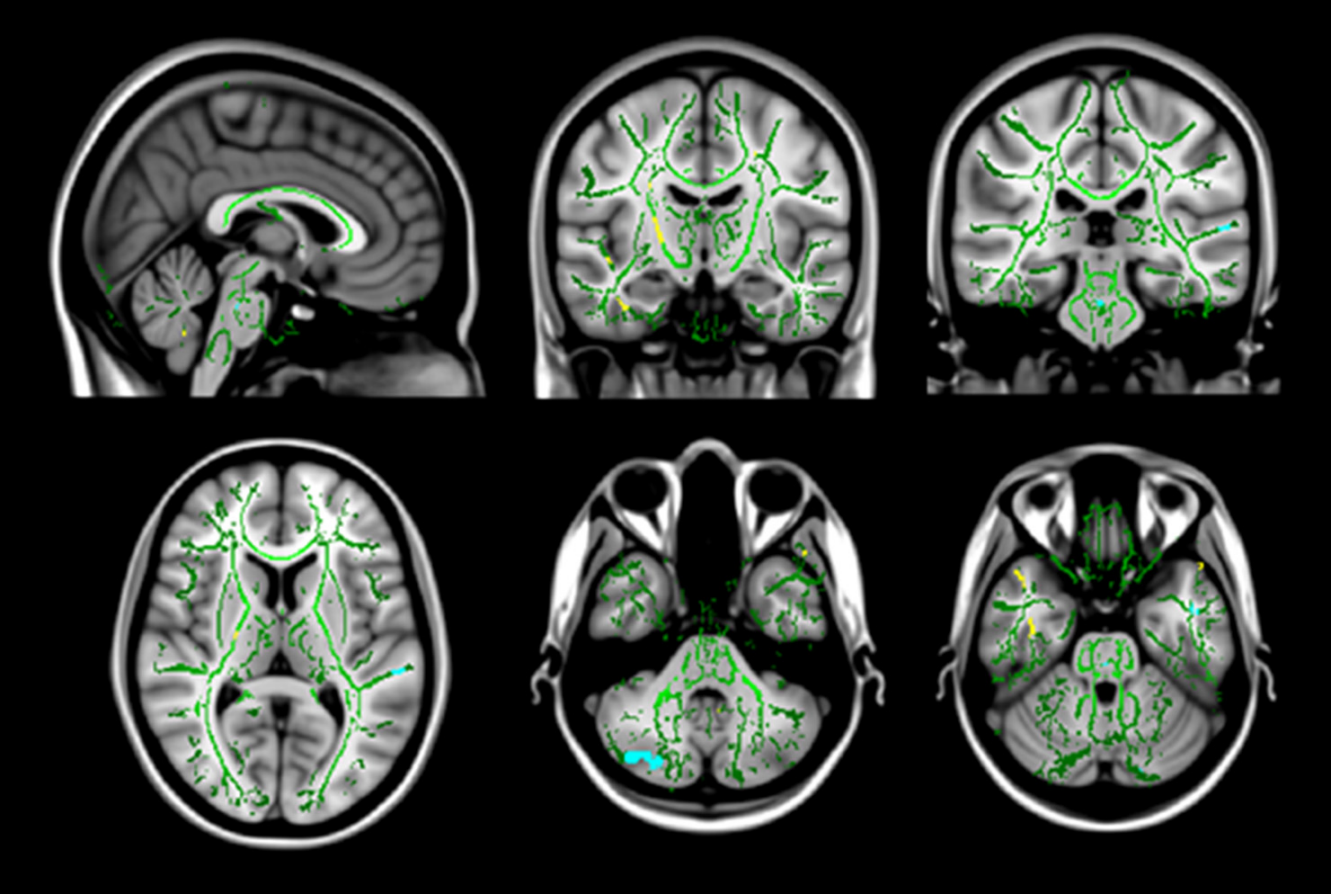
Changes in FA values in patients with RLS. Coronal, axial, and sagittal view of the mean b0 image with the overlayed common white matter skeleton (green), showing areas of significantly (*P* < 0.0001) increased (yellow) and decreased (blue) fractional anisotropy.

**Figure 2:**
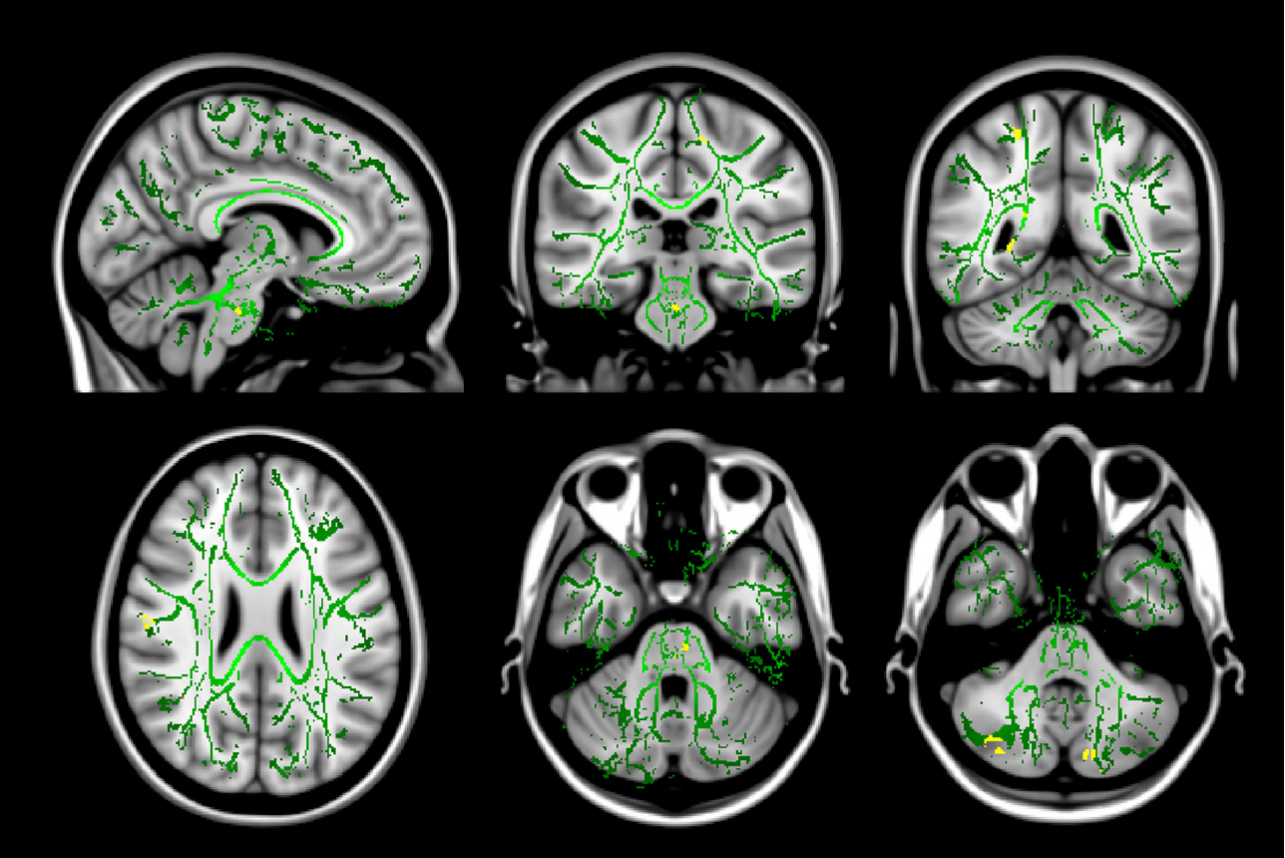
Changes in RD values in patients with RLS. Coronal, axial, and sagittal view of the mean b0 image with the overlayed common white matter skeleton (green), showing areas of significantly (*P* < 0.0001) increased (yellow) radial diffusivity.

### VBM results

Voxel-based morphometry did not show any areas of altered gray matter volume in patients with RLS as compared to healthy control subjects after correction for multiple comparisons.

## Discussion

Using DTI we could show altered diffusion properties reflecting microstructural or functional white matter changes mainly in the temporal subcortical white matter, the internal capsule, the pons, and the cerebellum of patients with iRLS. Voxel-based morphometry revealed no changes of gray matter volume associated with these white matter alterations.

### Sensorimotor pathways

Two earlier studies investigating patients with RLS using DTI revealed conflicting results.

Unrath et al. (2008) reported changes in FA bihemispherically in close proximity to the primary and associate motor and somatosensory cortices, in the right thalamus, motor projectional fibers, and adjacent to the left anterior cingulum and concluded that RLS is related to changes in the sensorymotor pathway. This hypothesis was also supported by a second study conducted by the same group using VBM (Unrath et al. 2007). However, other studies found no differences between patients with RLS and normal control subjects using DTI and TBSS (Rizzo et al. 2012) or VBM (Celle et al. 2010; Margariti et al. 2012). These conflicting results might be explained by methodological differences like the use of TBSS and the use of different software, as well as differences in clinical parameters including medication status, RLS severity or disease duration.

Our own results include changes of FA in the internal capsule which are in line with changes in the sensorimotor pathways in iRLS suggested by Unrath et al. (2008). Changes were located in the posterior limb of the internal capsule in an area which contains the pyramidal tract to the lower limb. These findings are in line with the clinical observations of symptoms that are accentuated in the legs.

However, we only discovered small areas of altered diffusion using DTI and no alterations of gray matter volume using VBM. Considering the normal results of another DTI (Rizzo et al. 2012) and several VBM studies (Celle et al. 2010; Margariti et al. 2012) it remains questionable if RLS is indeed associated with clear structural brain changes.

### Cerebellum

The areas of altered white matter diffusion properties in the cerebellum seem surprising at first. However, one of the few fMRI studies on patients with iRLS investigated changes in brain activation during sensory symptoms and periodic limb movements and found activation in the cerebellum (Bucher et al. 1997). The authors attributed these findings to an involvement of the cerebellum in sensory processing, which was also supported by a study on healthy subjects (Jueptner et al. 1995).

### Dopaminergic system

An involvement of the dopaminergic system in the pathophysiology of iRLS has been extensively discussed (Staedt et al. 1993, 1995a,b, 1996; Trenkwalder et al. 1999; Turjanski et al. 1999; Ruottinen et al. 2000; Eisensehr et al. 2001; Michaud et al. 2002; Tribl et al. 2004; Cervenka et al. 2006). This hypothesis is supported by the effect of dopaminergic medication on the RLS symptoms as well as results of PET and SPECT studies investigating pre- and postsynaptic and extrastriatal dopamine receptor binding (Turjanski et al. 1999; Ruottinen et al. 2000; Cervenka et al. 2006). However, other PET and SPECT studies are at variance, showing no alterations in the dopaminergic system (Trenkwalder et al. 1999; Eisensehr et al. 2001; Michaud et al. 2002; Tribl et al. 2004). These studies are also supported by normal results of histopathological studies examining the substantia nigra and the dopaminergic neurons of the hypothalamic A11 system (Earley et al. 2009).

In accordance with the two previous DTI studies (Unrath et al. 2008; Rizzo et al. 2012) and several VBM studies (Unrath et al. 2007; Celle et al. 2010; Connor et al. 2010; Comley et al. 2012; Margariti et al. 2012; Rizzo et al. 2012), we found no areas of altered white matter microstructure or function or gray matter volume in striatal areas. A possible involvement of the dopaminergic system in the pathophysiology of iRLS does therefore not seem to be associated with cerebral microstructural or functional changes detectable by DTI or VBM. Possible changes on the spinal level were not investigated in this study.

### Brain iron concentration and hypomyelination

Several studies revealed a reduced brain iron concentration in patients with RLS (Allen et al. 2001; Astrakas et al. 2008), which was not confirmed in other studies (Knake et al. 2010b; Margariti et al. 2012) and might only be present in patients with early-onset RLS (Earley et al. 2006). It has been shown that iron deficiency can be associated with hypomyelination in animals (Yu et al. 1986; Beard et al. 2003; Ortiz et al. 2004) and humans (Roncagliolo et al. 1998; Connor et al. 2010). One study described hypomyelination in patients with RLS associated with low ferritin and transferrin in the myelin fraction (Connor et al. 2010).

Changes in FA and RD as observed in this study can represent changes associated with myelination or glial cell morphology (Song et al. 2002, 2003). The multiple small areas of altered white matter diffusion properties in subcortical temporal, frontal, parietal and occipital regions, cerebellum, and internal capsule that include diverse brain regions and systems might therefore reflect altered myelination that might possibly be related to changes in brain iron content.

### Gray matter volume

The white matter changes observed in this study were not associated with any changes in gray matter volume. Several studies have investigated gray matter volume using VBM with conflicting results. Some authors describe gray matter changes in discordant brain regions including the pulvinar (Etgen et al. 2005), the primary somatosensory cortex bilaterally and the left primary motor area (Unrath et al. 2007), the callosal body, anterior cingulum and precentral gyrus (Connor et al. 2010) and the ventral hippocampus and middle orbitofrontal gyrus (Hornyak et al. 2007). The discordant results might, in part, be due to differences in clinical parameters like medication status, duration of RLS or RLS symptoms or differences in methodology like the use of an optimized VBM protocol or different methods for coregistration. Also, in several studies, the brain regions described were only significant when an uncorrected significance level was used (Etgen et al. 2005; Unrath et al. 2007) and did not survive correction for multiple comparisons (Unrath et al. 2007). The lack of a significant overlap of the altered brain regions and the numerous studies that found no significant differences in gray matter volume (Celle et al. 2010; Comley et al. 2012; Margariti et al. 2012; Rizzo et al. 2012) imply that there are no constant changes in gray matter volume that can be measured by VBM. This hypothesis is also confirmed by our study results.

### Conclusion and open questions

In this study, we investigated white and gray matter changes in patients with iRLS using DTI and VBM. To our knowledge, there is only one earlier study which applied both methods simultaneously and showed normal results when using TBSS and a corrected threshold at *P* < 0.05. There were small areas of altered DTI parameters only when the authors used a less restrictive threshold that was not corrected for multiple comparisons (Rizzo et al. 2012). We discovered small areas of microstructural or functional white matter changes in subcortical regions, the cerebellum and the internal capsule that were not associated with any changes in gray matter volume. The areas of altered white matter diffusion properties were only small and it remains unclear if they really represent microstructural or functional changes associated with iRLS.

This study focuses on alterations in the brain that are associated with iRLS. Possible alterations in the spinal cord or peripheral nervous system that might also contribute to the pathophysiology of iRLS are not investigated in this study and require future research, especially in the light of the inconsistent brain changes.

Limitations of the present as well as earlier (Unrath et al. 2008; Rizzo et al. 2012) DTI studies include the use of dopaminergic medication, which has been shown to affect brain structure and microstructure (Hagino et al. 1998; Salgado-Pineda et al. 2006). The dopaminergic medication might on one hand have caused the diffusion changes found in the present and earlier studies or might on the other hand have obscured other microstructural or functional changes associated with RLS. Studies on drug-naïve patients are warranted.

Other factors influencing the results could be disease duration and the severity of the symptoms. In this study, disease duration was relatively short as compared to an earlier DTI study (Unrath et al. 2008) and several VBM studies (Unrath et al. 2007; Comley et al. 2012). Longer disease duration might be associated with more pronounced changes of brain microstructure. On the other hand, this study investigated only patients with moderate to severe symptoms on the CGI and showed that even in this group of strongly affected patients only small areas of altered diffusion parameters can be found.

## Conflict of Interests

None declared.

## References

[b1] Allen RP, Barker PB, Wehrl F, Song HK, Earley CJ (2001). MRI measurement of brain iron in patients with restless legs syndrome. Neurology.

[b2] Allen RP, Picchietti D, Hening WA, Trenkwalder C, Walters AS, Montplaisi J (2003). Restless legs syndrome: diagnostic criteria, special considerations, and epidemiology - A report from the restless legs syndrome diagnosis and epidemiology workshop at the National Institutes of Health. Sleep Med.

[b3] Andersson JLR, Jenkinson M, Smith S (2007). http://www.fmrib.ox.ac.uk/analysis/techrep.

[b4] Astrakas L, Konitsiotis S, Margariti P, Tsouli S, Tzarouhi L, Argyropoulou MI (2008). T2 relaxometry and fMRI of the brain in late-onset restless legs syndrome. Neurology.

[b5] Basser PJ (1997). New histological and physiological stains derived from diffusion-tensor MR images. Ann. N. Y. Acad. Sci.

[b6] Beard JL, Wiesinger JA, Connor JR (2003). Pre- and postweaning iron deficiency alters myelination in Sprague-Dawley rats. Dev. Neurosci.

[b7] Beaulieu C, Allen PS (1994). Determinants of anisotropic water diffusion in nerves. Magn. Reson. Med.

[b8] Brenneis C, Brandauer E, Frauscher B, Schocke M, Trieb T, Poewe W (2005). Voxel-based morphometry in narcolepsy. Sleep Med.

[b9] Bucher SF, Seelos KC, Oertel WH, Reiser M, Trenkwalder C (1997). Cerebral generators involved in the pathogenesis of the restless legs syndrome. Ann. Neurol.

[b10] Celle S, Roche F, Peyron R, Faillenot I, Laurent B, Pichot V (2010). Lack of specific gray matter alterations in restless legs syndrome in elderly subjects. J. Neurol.

[b11] Cervenka S, Palhagen SE, Comley RA, Panagiotidis G, Cselenyi Z, Matthews JC (2006). Support for dopaminergic hypoactivity in restless legs syndrome: a PET study on D2-receptor binding. Brain.

[b12] Comley RA, Cervenka S, Palhagen SE, Panagiotidis G, Matthews JC, Lai RY (2012). A comparison of gray matter density in restless legs syndrome patients and matched controls using voxel-based morphometry. J. Neuroimaging.

[b13] Connor JR, Ponnuru P, Lee BY, Yang Q, Podskalny GD, Alam S (2010). Postmortem and imaging based analyses reveal CNS hypomyelination in restless legs syndrome. Neurology.

[b14] Douaud G, Smith S, Jenkinson M, Behrens T, Johansen-Berg H, Vickers J (2007). Anatomically related grey and white matter abnormalities in adolescent-onset schizophrenia. Brain.

[b15] Earley CJ, Barker PB, Horska A, Allen RP (2006). MRI-determined regional brain iron concentrations in early- and late-onset restless legs syndrome. Sleep Med.

[b16] Earley CJ, Allen RP, Connor JR, Ferrucci L, Troncoso J (2009). The dopaminergic neurons of the A11 system in RLS autopsy brains appear normal. Sleep Med.

[b17] Eisensehr I, Wetter TC, Linke R, Noachtar S, von Lindeiner H, Gildehaus FJ (2001). Normal IPT and IBZM SPECT in drug-naive and levodopa-treated idiopathic restless legs syndrome. Neurology.

[b18] Etgen T, Draganski B, Llg C, Schroder M, Geisler P, Hajak G (2005). Bilateral thalamic gray matter changes in patients with restless legs syndrome. NeuroImage.

[b19] Glenn OA, Henry RG, Berman JI, Chang PC, Miller SP, Vigneron DB (2003). DTI-based three-dimensional tractography detects differences in the pyramidal tracts of infants and children with congenital hemiparesis. J. Magn. Reson. Imaging.

[b20] Good CD, Johnsrude IS, Ashburner J, Henson RNA, Friston KJ, Frackowiak RSJ (2001). A voxel-based morphometric study of ageing in 465 normal adult human brains. NeuroImage.

[b21] Hagino H, Tabuchi E, Kurachi M, Saitoh O, Sun YJ, Kondoh T (1998). Effects of D-2 dopamine receptor agonist and antagonist on brain activity in the rat assessed by functional magnetic resonance imaging. Brain Res.

[b22] Hornyak M, Ahrendts JC, Spiegelhalder K, Riemann D, Voderholzer U, Feige B (2007). Voxel-based morphometry in unmedicated patients with restless legs syndrome. Sleep Med.

[b23] Jueptner M, Rijntjes M, Weiller C, Faiss JH, Timmann D, Mueller SP (1995). Localization of a cerebellar timing process using pet. Neurology.

[b24] Kaufmann C, Schuld A, Pollmacher T, Auer DP (2002). Reduced cortical gray matter in narcolepsy: preliminary findings with voxel-based morphometry. Neurology.

[b25] Knake S, Belke M, Menzler K, Pilatus U, Eggert KM, Oertel WH (2010a). In vivo demonstration of microstructural brain pathology in progressive supranuclear palsy: a DTI study using TBSS. Mov. Disord.

[b26] Knake S, Heverhagen JT, Menzler K, Keil B, Oertel WH, Stiasny-Kolster K (2010b). Normal regional brain iron concentration in restless legs syndrome measured by MRI. Nat. Sci. Sleep.

[b27] Margariti P, Astrakas L, Tsouli S, Hadjigeorgiou G, Konitsiotis S, Argyropoulou MI (2012). Investigation of unmedicated early onset restless legs syndrome by voxel-based morphometry, T2 relaxometry, and functional MR imaging during the night-time hours. Am. J. Neuroradiol.

[b28] Menzler K, Belke M, Wehrmann E, Krakow K, Lengler U, Jansen A (2011). Men and women are different: diffusion tensor imaging reveals sexual dimorphism in the microstructure of the thalamus, corpus callosum and cingulum. NeuroImage.

[b29] Menzler K, Belke M, Unger M, Ohletz T, Keil B, Heverhagen J (2012). DTI reveals hypothalamic and brainstem white matter lesions in patients with idiopathic narcolepsy. Sleep Med.

[b30] Michaud M, Soucy JP, Chabli A, Lavigne G, Montplaisir J (2002). SPECT imaging of striatal pre- and postsynaptic dopaminergic status in restless legs syndrome with periodic leg movements in sleep. J. Neurol.

[b31] Ortiz E, Pasquini JM, Thompson K, Felt B, Butkus G, Beard J (2004). Effect of manipulation of iron storage, transport, or availability on myelin composition and brain iron content in three different animal models. J. Neurosci. Res.

[b32] Paulus W, Dowling P, Rijsman R, Stiasny-Kolster K, Trenkwalder C, de Weerd A (2007). Pathophysiological concepts of restless legs syndrome. Mov. Disord.

[b33] Pierpaoli C, Basser PJ (1996). Toward a quantitative assessment of diffusion anisotropy. Magn. Reson. Med.

[b34] Rizzo G, Manners D, Vetrugno R, Tonon C, Malucelli E, Plazzi G (2012). Combined brain voxel-based morphometry and diffusion tensor imaging study in idiopathic Restless Legs Syndrome patients. Eur. J. Neurol.

[b35] Roncagliolo M, Garrido M, Walter T, Peirano P, Lozoff B (1998). Evidence of altered central nervous system development in infants with iron deficiency anemia at 6 mo: delayed maturation of auditory brainstem responses. Am. J. Clin. Nutr.

[b36] Ruottinen HM, Partinen M, Hublin C, Bergman J, Haaparanta M, Solin O (2000). An FDOPA PET study in patients with periodic limb movement disorder and restless legs syndrome. Neurology.

[b37] Salat DH, Tuch DS, van der Kouwe AJ, Greve DN, Pappu V, Lee SY (2008). White matter pathology isolates the hippocampal formation in Alzheimer’s disease. Neurobiol. Aging.

[b38] Salgado-Pineda P, Delaveau P, Falcon C, Blin O (2006). Brain T1 intensity changes after levodopa administration in healthy subjects: a voxel-based morphometry study. Br. J. Clin. Pharmacol.

[b39] Smith SM, Jenkinson M, Woolrich MW, Beckmann CF, Behrens TE, Johansen-Berg H (2004). Advances in functional and structural MR image analysis and implementation as FSL. NeuroImage.

[b40] Song SK, Sun SW, Ramsbottom MJ, Chang C, Russell J, Cross AH (2002). Dysmyelination revealed through MRI as increased radial (but unchanged axial) diffusion of water. NeuroImage.

[b41] Song SK, Sun SW, Ju WK, Lin SJ, Cross AH, Neufeld AH (2003). Diffusion tensor imaging detects and differentiates axon and myelin degeneration in mouse optic nerve after retinal ischemia. NeuroImage.

[b42] von Spiczak S, Whone AL, Hammers A, Asselin MC, Turkheimer F, Tings T (2005). The role of opioids in restless legs syndrome: an [C-11]diprenorphine PET study. Brain.

[b43] Spiegelhalder K, Feige B, Paul D, Riemann D, van Elst LT, Seifritz E (2008). Cerebral correlates of muscle tone fluctuations in restless legs syndrome: a pilot study with combined functional magnetic resonance imaging and anterior tibial muscle electromyography. Sleep Med.

[b44] Staedt J, Stoppe G, Kogler A, Munz D, Riemann H, Emrich D (1993). Dopamine-D2 receptor alteration in patients with periodic movements in sleep (Nocturnal Myoclonus). J. Neural. Transm.

[b45] Staedt J, Stoppe G, Kogler A, Riemann H, Hajak G, Munz DL (1995a). Nocturnal myoclonus syndrome (periodic movements in sleep) related to central dopamine D2-receptor alteration. Eur. Arch. Psychiatry Clin. Neurosci.

[b46] Staedt J, Stoppe G, Kogler A, Riemann H, Hajak G, Munz DL (1995b). Single-photon emission tomography (SPET) imaging of dopamine D-2 receptors in the course of dopamine replacement therapy in patients with nocturnal myoclonus syndrome (NMS). J. Neural. Transm.

[b47] Staedt J, Stoppe G, Kogler A, Riemann H, Hajak G, Rodenbeck A (1996). [123I]IBZM SPET analysis of dopamine D2 receptor occupancy in narcoleptic patients in the course of treatment. Biol. Psychiatry.

[b48] Trenkwalder C, Walters AS, Hening WA, Chokroverty S, Antonini A, Dhawan V (1999). Positron emission tomographic studies in restless legs syndrome. Mov. Disord.

[b49] Tribl GG, Asenbaum S, Happe S, Bonelli RM, Zeitlhofer J, Auff E (2004). Normal striatal D-2 receptor binding in idiopathic restless legs syndrome with periodic leg movements in sleep. Nucl. Med. Commun.

[b50] Turjanski N, Lee AJ, Brooks DJ (1999). Striatal dopaminergic function in restless legs syndrome - F-18-dopa and C-11-raclopride PET studies. Neurology.

[b51] Unger MM, Belke M, Menzler K, Heverhagen J, Keil B, Stiasny-Kolster K (2010). Diffusion tensor imaging in idiopathic REM sleep behaviour disorder (iRBD) reveals microstructural changes in the brainstem, the olfactory cortex and other brain regions. Sleep.

[b52] Unrath A, Kassubek J (2006). Symptomatic restless leg syndrome after lacunar stroke: a lesion study. Mov. Disord.

[b53] Unrath A, Juengling FD, Schork M, Kassubek J (2007). Cortical grey matter alterations in idiopathic restless legs syndrome: an optimized voxel-based morphometry study. Mov. Disord.

[b54] Unrath A, Mueller HP, Ludolph AC, Riecker A, Kassubek J (2008). Cerebral white matter alterations in idiopathic restless legs syndrome, as measured by diffusion tensor imaging. Mov. Disord.

[b55] Yu GSM, Steinkirchner TM, Rao GA, Larkin EC (1986). Effect of prenatal iron-deficiency on myelination in rat pups. Am. J. Pathol.

